# Effects of ascorbic acid on sperm motility, viability, acrosome reaction and DNA integrity in teratozoospermic samples

**Published:** 2014-02

**Authors:** Hamed Fanaei, Samira Khayat, Iman Halvaei, Vahid Ramezani, Yaser Azizi, Amir Kasaeian, Jalal Mardaneh, Mohammad Reza Parvizi, Maryam Akrami

**Affiliations:** 1 *Pregnancy Health Research Center, Zahedan University of Medical Sciences, Zahedan, Iran.*; 2 *Department of Physiology, School of Medicine, Zahedan University of Medical Sciences, Zahedan, Iran.*; 3 *Nursing and Midwifery Faculty, Shahid Beheshti University of Medical Sciences, Tehran, Iran.*; 4 *Research and Clinical Center for Infertility, Shahid Sadoughi University of Medical Sciences, Yazd, Iran.*; 5 *Department of Pharmaceutics, Faculty of Pharmacy, Shahid Sadoughi University of Medical Sciences, Yazd, Iran.*; 6 *Department of Physiology, School of Medicine, Tehran University of Medical Sciences, Tehran, Iran.*; 7 *Non-Communicable Diseases Research Center, Endocrinology and Metabolism Population Sciences Institute, Tehran University of Medical Sciences, Tehran, Iran.*; 8 * Prof. Alborzi Clinical Microbiology Research Center, Shiraz University of Medical Sciences, Shiraz, Iran.*; 9 *Kashan University of Medical Sciences, Kashan, Iran.*

**Keywords:** *Ascorbic acid*, *Teratozoospermic sperm*, *Acrosome reaction*, *DNA fragmentation*, *Oxidative stress*

## Abstract

**Background:** Oxidative stress in teratozoospermic semen samples caused poor assisted reproductive techniques (ART) outcomes. Among antioxidants, ascorbic acid is a naturally occurring free radical scavenger and as such its presence assists various other mechanisms in decreasing numerous disruptive free radical processes.

**Objective: **The main goal of this study was to evaluate potential protective effects of ascorbic acid supplementation during in vitro culture of teratozoospermic specimens.

**Materials and Methods:** Teratozoospermic semen samples that collected from 15 volunteers were processed, centrifuged and incubated at 37^o^C until sperm swimmed-up. Supernatant was divided into four groups and incubated at 37^o^C for one hour under different experimental conditions: Control, 10 µm A23187, 600µm ascorbic acid and 10 µm A23187+600 µm ascorbic acid. After incubation sperm motility, viability, acrosome reaction, DNA damage and malondialdehyde levels were evaluated.

**Results:** Our results indicated that after one hour incubation, ascorbic acid significantly reduced malondialdehyde level in ascorbic acid group (1.4±0.11 nmol/ml) compared to control group (1.58±0.13 nmol/ml) (p<0.001). At the end of incubation, progressive motility and viability in ascorbic acid group (64.5±8.8% and 80.3±6.4%, respectively) were significantly (p<0.05 and p<0.001, respectively) higher than the control group (54.5±6.8% and 70.9±7.3%, respectively). A23187 significantly (p<0.0001) increased acrosome reaction in A23187 group (37.3±5.6%) compared to control group (8.5±3.2%) and this effect of A23187 attenuated by ascorbic acid in ascorbic acid+A23187 group (17.2±4.4%). DNA fragmentation in ascorbic acid group (20±4.1%) was significantly (p<0.001) lower than controls (28.9±4.6%).

**Conclusion:** In vitro ascorbic acid supplementation during teratozoospermic semen processing for ART could protect teratozoospermic specimens against oxidative stress, and it could improve ART outcome.

## Introduction

Most of men with teratozoospermia suffer from infertility due to defects in their spermatogenesis process (1-3). Teratozoospermia are characterized by abnormal shape sperm in semen which are expressed in more than 70% of sperm cells in each ejaculation (4-6). In physiological conditions, the successful spontaneous pregnancy is highly depended, on passing the various crucial steps including; oocyte fertilization, syngamy of male and female pronuclei, embryonic cleavage, genomic activation, blastulation and implantation (7, 8). Therefore, the sperm disability of most of the teratozoospermic patients in order to produce natural pregnancies may be related to their lack of ability to pass some mentioned steps. Several studies have been reported that teratozoospermia are associated with various disorders such as; poor rates of fertilization, development of embryo, implantation and maintenance of pregnancy and even childhood cancer (1, 7, 9, 10). 

Nowadays, most of men with teratozoospermic need assisted reproductive techniques (ART) (7, 11). They usually undergoing in vitro fertilization (IVF) and in severe cases use intracytoplasmic sperm injection (ICSI) for conception; nevertheless their ART outcomes are poor (7, 8, 11-14). One of the main reasons for teratozoospermic samples malfunctions is high rates of reactive oxygen species (ROS) production (1, 3). Exact sources of high ROS production in teratozoospermia are not clear, but some studies have suggested that mitochondria, cytoplasm residues and NADPH oxidase 5 may be responsible (1, 15).

In physiological conditions, sperm cells produce ROS at low and controlled levels and these ROS, as second messengers, have many roles during various sperm functions (e.g. capacitation, acrosome reaction and fertilization) (16). While excessive ROS generation or low antioxidants concentration in semen, induces oxidative stress (OS), which results in production of oxygen-derived oxidants, and in turn has deleterious effects on spermatozoa (1, 17). OS may damage many sperm functions which is depended on plasma membrane (e.g. motility, viability, acrosome reaction and sperm-oocyte fusion) and DNA integrity (e.g. fertilization, development of embryo, implantation and maintenance of pregnancy) (1, 16-18). 

To neutralize the effects of OS and promote sperm quality, it has been shown that antioxidants have beneficial effects in treating male infertility (19, 20). Ascorbic acid is an effective water-soluble ROS scavenger with high potency (19). Therefore, addition of ascorbic acid to sperm washing medium can enhance sperm performance by neutralizing ROS. Animal studies demonstrated that ascorbic acid can improve sperm parameters (21-24). In addition, in vivo human studies have proved the beneficial effects of oral administration of ascorbic acid on sperm concentration, motility and DNA damage levels in oligo-astheno-teratozoospermic patients (25, 26). 

Therefore, in this investigation we tried to examine the impacts of ascorbic acid addition to teratozoospermic sperm medium on sperm motility, viability, acrosome reaction and DNA integrity after one hour incubation at 37^o^C. 

## Materials and methods

This work was a case control study and the Human Research Ethics Committees at Tehran University of Medical Sciences approved all experiments. The present study was performed at Department of Physiology between April 2012 and January 2013. Written informed consent was obtained from all participants for the study. All chemicals were obtained from Sigma-Aldrich, unless otherwise stated.

**Semen samples**

The specimens were entered into study chronologically. Teratozoospermic semen samples were obtained from 15 males between 20 and 35 years old undergoing routine semen analysis for male infertility. If the teratozoospermic volunteers used any drugs, vitamins, alcohol or tobacco, they were excluded from the study. Semen samples were collected in sterile containers after 3-5 days of sexual abstinence. 

The samples were liquefied at room temperature (22-24^o^C) for 30 minutes. Then, semen analysis was performed and samples with >70% abnormal shapes were classiﬁed as teratozoospermia. Other semen characteristics were normal in all selected samples according to 2010 WHO semen parameters criteria (5). 

**Semen preparation and treatment**

Semen samples were washed with Ham’s F-10 medium and centrifuged at 500 g for 10 minutes and then seminal plasma was removed. Motile sperm fractions were retrieved from the samples using swim-up technique in Ham’s F-10 medium containing 5% human serum albumin and incubated at 37^o^C with 5% CO_2_. Sperm motility, viability, DNA fragmentation and malondialdehyde (MDA) levels were evaluated before incubation. 

After one hour of incubation, the supernatant containing motile sperm was collected. Supernatant was divided into four groups, in each group concentration was adjusted to 2×10^6^ sperm/ml. After that, groups 1-4 were incubated for one hour in 37^o^C and 5% CO_2_ with Ham’s F-10 solution as control group, 10 µm A23187, 600 µm ascorbic acid and 10 µm A23187+600 µm ascorbic acid, respectively. Motility, viability, acrosome reaction, DNA fragmentation and MDA levels were subsequently assessed.

**Oxidative stress assay by measuring MDA levels**

MDA levels were measured by using the Thiobarbituric Acid (TBA) method (27,28). Sample from each group was centrifuged at 3000 rpm for 20 minutes and then supernatant was separated. After that, 100 µl of supernatant was added to100 µl of 1% TBA reagent and 100 µl of 20% trichloroacetic acid. They were mixed and incubated at 100^o^C (in a water bath) for 80 minutes. After cooling on ice, samples were centrifuged at 3000 rpm for 20 minutes and supernatant absorbance was read by a spectrophotometer at 532 nm.

**Assessment of sperm motility and viability**

In all samples, a standard sperm motility analysis was performed according to 2010 WHO guidelines. Immediately after incubation, 10 µl of each group was placed on a microscope slide and covered with a 22×22 mm cover-slip. Then, preparation of the slide was assessed under 400× magnification using light microscopy. At least 200 spermatozoa were examined in each evaluation. 

Motility of each sperm was graded in three clusters: progressive motility, non-progressive motility and immotility that were reported as percentage. Sperm viability was evaluated by eosin Y staining. After incubation, 20 µl of each group was mixed with 20 µl of eosin Y solution on a glass microscope slide and was monitored using light microscopy at a magnification of 400× to evaluate the percentage of viable sperm. Viable sperm cells were remained colorless, while dead sperm cells were stained red. At least 200 spermatozoa were examined in each evaluation.

**Evaluation of the acrosomal status**

The calcium ionophore A23187 was used as pharmacological acrosome reaction inducer. Staining with ﬂuorescein isothiocyanate-conjugated Pisum sativum (FITC-PSA) was performed according to Cross et al method (1). Briefly, an aliquot (30 µl) of sperm was smeared onto glass slide and allowed to air-dry. The spermatozoa on the slide were then permeabilised by methanol for 30 seconds at room temperature, washed with double-distilled water, dried at room temperature, incubated for 30 minutes with 50 µl of FITC-PSA at room temperature in a moisture chamber and were washed with double-distilled water. 

The slide was then covered with a mounting ﬂuid (glycerol) and a cover glass. By means of a ﬂuorescence microscope (Olympus BX51, Tokyo, Japan) at least 200 sperm were differentiated blindly at 1000× magniﬁcation according to the ﬂuorescence pattern of their acrosomes. Sperm with green ﬂuorescence in the acrosomal region were scored as acrosome intact, whereas those with no green ﬂuorescence in the acrosomal region or only ﬂuorescence of the equatorial segment were considered as acrosome-reacted.

**Assessment of DNA fragmentation**

Sperm DNA damage was evaluated by terminal deoxynucleotidyl transferase mediated dUTP nick end labeling (TUNEL) assay. The TUNEL assay was performed according to the manufacturer’s guidelines (In-Situ Cell Death Detection Kit, Fluorescein; Roche Diagnostics GmbH, Mannheim, Germany). Briefly, a droplet of the sperm suspension of each group was spread out over slide; thereafter, air dried smeared slides were fixed in 4% paraformaldehyde at room temperature and rinsed in PBS (pH=7.4) and then permeabilized with 2% Triton X-100.

Terminal deoxytransferase (TdT)-labelled nucleotide mixture was added to each slide and incubated in a humidified chamber in the darkness under 37^o^C for 60 minutes. Later, slides were rinsed twice in PBS. Negative controls without TdT enzyme were operated in each duplicate. Leastwise, 200 sperm per slide were assessed using ﬂuorescence microscopy (Olympus BX51, Tokyo, Japan) at 1000× magniﬁcations by the same examiner. The percentage of cells with green ﬂuorescence (TUNEL positive) was determined.

**Statistical analysis**

Statistical analysis was carried out using the software package SPSS 16. Results are expressed as Mean±SD. The significance of the difference between the treatments was assessed using nonparametric Mann-Whitney Test. Differences were considered to be statistically significant when p<0.05.

## Results

**MDA levels**

**Figure 1:**
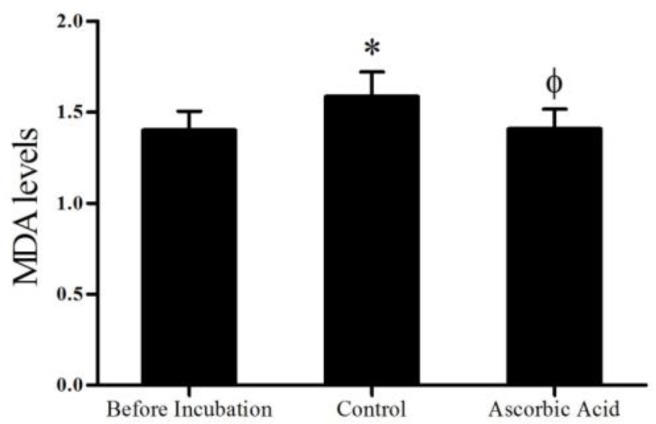
Effects of ascorbic acid on MDA levels one hour after incubation (mean±SD). *Signiﬁcant difference (p<0.001) compared to before incubation group. Φ Signiﬁcant difference (p<0.001) with control group

The values for MDA levels are shown in figure 1. After one hour incubation, MDA level in the control group (1.58±0.13 nmol/ml) was significantly higher (p<0.001) than before incubation (1.39±0.1 nmol/ml). On the other hand, results of MDA level of ascorbic acid group (1.4±0.11 nmol/ml) showed that ascorbic acid significantly (p<0.001) reduced MDA level in this group compared to control group. Ascorbic acid group had no significant difference compared to before incubation group.

**Sperm motility and viability**

Progressive and non-progressive motility data are presented in table I. Analysis of data was indicated that progressive motility after one hour incubation in control group (54.5±6.8) significantly (p<0.0001) reduced compared to before incubation (69.1±5.5%). The progressive motility in the ascorbic acid group was signiﬁcantly (p<0.05) higher (64.5±8.8) than control group. On the other hand, non-progressive motility in the control group was significantly higher (22.9±5) (p<0.0001) compared to before incubation (13.8±3.6) (Table I). Ascorbic acid treatment led to significant decrease in the non-progressive motility percent up to 14.9±4.6 (p<0.001) in comparison with control group (Table I). The values for sperm viability are demonstrated in table I. Analysis of data showed that the sperm viability in before incubation group (84.3±6.3) (p<0.0001) and ascorbic acid (80.3±6.4) (p<0.001) groups were significantly higher than control group (70.9±7.3).

**Acrosome reaction**

**Figure 2:**
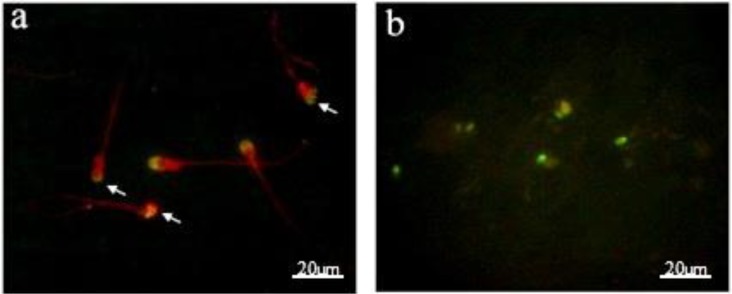
Detection of (a) acrosomal reaction by FITC-PSA staining method. The arrows show acrosome reacted sperm cells. (b) DNA damage by TUNEL staining method. The greenish sperm cells are DNA fragmented

**Figure 3:**
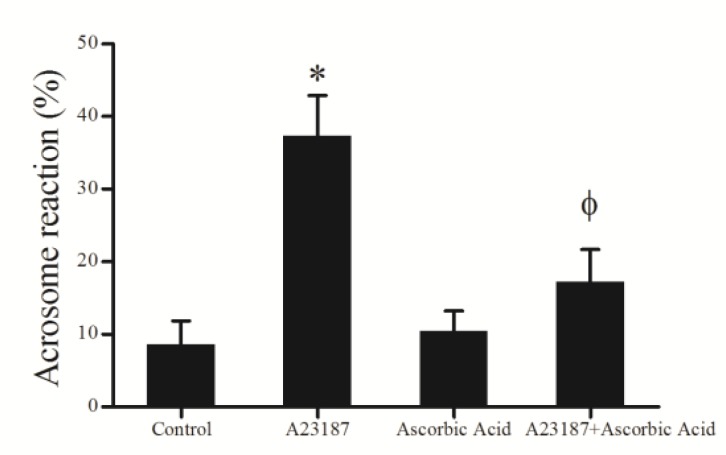
Effects of A23187, ascorbic acid and A23187 in combination with ascorbic acid on acrosome reaction of teratozoospermic samples one hour after incubation (mean±SD). *Signiﬁcant difference (p<0.0001) compared to other groups. ϕ Signiﬁcant difference (p<0.001) compared to ascorbic acid group

Figure 2a demonstrates examples of acrosomal status and acrosomal reaction data which are presented in figure 3. Administration of A23187 in A23187 group induced a significant increase (37.3±5.6) (p<0.0001) in acrosome reaction; whereas ascorbic acid in ascorbic acid group (10.4±2.7) did not induce significant (p>0.05) changes in acrosome reaction when compared to control group (8.5±3.2). On the other hand, comparison of ascorbic acid + A23187 group (17.2±4.4) with A23187 group showed that ascorbic acid was attenuated the effects of A23187 on acrosome reaction, while acrosome reaction in ascorbic acid + A23187 group was significantly higher (p<0.001) than ascorbic acid group.

**DNA fragmentation**

**Figure 4:**
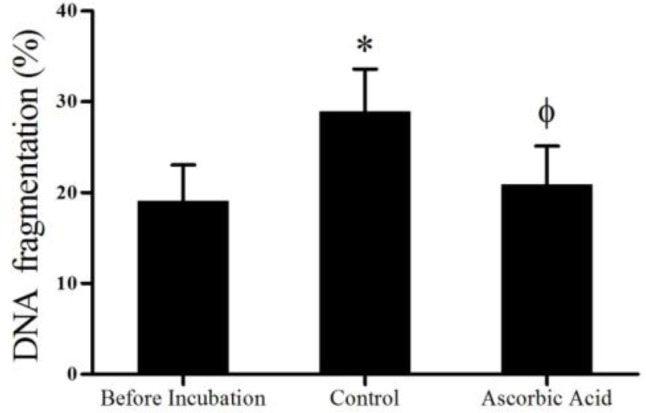
Effects of ascorbic acid on DNA integrity of teratozoospermic samples one hour after incubation (mean±SD). *Signiﬁcant difference (p<0.0001) compared to before incubation group. ϕ Signiﬁcant difference (p<0.001) compared to control group

Data concerning DNA damage are presented in figures 2b and 4. After one hour incubation, DNA fragmentation in control group (28.9±4.6) (p<0.0001) significantly was increased compared to before incubation (19.1±3.9). On the other hand, comparison of ascorbic acid group (20±4.1) with control group showed that ascorbic acid significantly (p<0.001) prevented the increase of DNA damage.

**Table I:** Effects of ascorbic acid on progressive motility, non-progressive motility and viability of teratozoospermic samples in different groups after 1 hour of incubation (mean±SD)

	**Before incubation (%)**	**Control (%)** a	**Ascorbic acid (%)**
Progressive motility	69.1 ± 5.5	54.5 ± 6.8	64.5 ± 8.8 b
Non-progressive motility	13.8 ± 3.6	22.9 ± 5	14.9 ± 4.6 c
Viability	84.3± 6.3	70.9 ± 7.3	80.3 ± 6.4 d

a Signiﬁcant difference (p<0.0001) compared to before incubation group.

b Signiﬁcant difference (p<0.05) compared to control group.

c Signiﬁcant difference (p<0.001) compared to control group.

d Signiﬁcant difference (p<0.001) compared to control group.

## Discussion

Our results indicated that ascorbic acid can protect teratozoospermia against ROS, and also improve sperm motility, viability and DNA integrity during incubation in teratozoospermic samples. But, there were no ameliorative effect of ascorbic acid on acrosome reaction and in fact, it may reduce acrosome reaction that was induced by A23187. As previously reported, the maintenance of sperm functions (e.g. motility, viability and acrosome reaction) and structures (e.g. plasma and mitochondrial membranes and DNA integrity) after ejaculation are important for success in ART and even natural pregnancy (29, 30).

OS is one of the main reasons of sperm quality loss after ejaculation (31). In physiological conditions, sperm quality maintenance is highly depended on balance in ROS levels around the sperm which is controlled by secretion of various antioxidants along the entire reproductive tract. But, after ejaculation, antioxidants defense slowly will eliminate around the sperm, and on the another hand, ROS production continues during sperm metabolism and causes accumulation of the excessive amounts of ROS and induce OS condition (32, 33). 

The relationship between diminished sperm quality and OS may be resulted a series of cascade events that cause a fall in intracellular ATP levels, release of different apoptogenic factors (eg, pro-caspases, cytochrome C, and apoptosis inducing factor) from mitochondria into the cytosole through disruption of mitochondrial membrane, inactivation of some biochemical pathways, enzyme dysfunction, disturbed axonemal protein phosphorylation, increased membrane permeability and generation of spermicidal products, which have adverse effects on the sperm functions (1, 16). 

In general, sperm quality reduces after ejaculation. This sperm quality reduction is troublesome for ART, particularly in cases with poor initial sperm quality, such as teratozoospermia. Compared with normal condition, in men with teratozoospermia, control of spermiogenesis is less efficient and consequently is resulted in production of numerous spermatozoa with deformities and malfunctions. Moreover, sperm of teratozoospermic specimens can produce high levels of ROS when compared to normal sperm (1). Therefore, this high ROS production accelerates reduction of sperm quality in teratozoospermia after ejaculation. Concurrently, other studies have shown that teratozoospermic semen has a shorter lifespan than normal semen and its quality noticeably reduces after ejaculation (1). 

Therefore, in the case of teratozoospermia, preservation of sperm quality after ejaculation is very important for ART success. Ascorbic acid is a well-known water-soluble antioxidant that reacts with ROS and thereby protects cell components (e.g. proteins, lipids and nucleic acids) against oxidative damages (34-36). In the present study, supplementation of the teratozoospermic sperm medium with ascorbic acid induced signiﬁcant improvements in MDA level, progressive motility, viability and DNA integrity of sperm from teratozoospermic specimens. Ascorbic acid may diminish ROS induced DNA fragmentation, recycle inactive vitamin E, and reduce lipid peroxidation (1).

Our result indicated that ascorbic acid did not improve percentage of acrosome reacted sperm cells and in fact it was decreased when induced by A23187. Previous studies have shown a steady rise of ROS production by teratozoospermia (1, 37). MDA is produced as a byproduct of lipid peroxidation during OS. MDA level measurement is a diagnostic tool in order to evaluate the quantity of lipid peroxidation level in cells. So it can apply as an indicator of OS and lipid peroxidation in sperm. In agreement with previous findings, our results showed that MDA level was increased after one hour in teratozoospermic sperm medium and ascorbic acid addition to medium prevented the further increase of MDA concentration. It is noteworthy that other studies have been reported that excessive ROS production in teratozoospermic semen samples is concurrent with significant decreases in antioxidants concentrations (38, 39). 

Low sperm quality of teratozoospermic samples after ejaculation can be the result of defective spermatogenesis, excessive ROS production and low antioxidants concentration. In teratozoospermia, there are no relevant data concerning in vitro effect of ascorbic acid supplementation, to which our findings could be compared. In agreement with our results, other studies have shown the potential protective effects of ascorbic acid on human sperm DNA integrity against oxidative damage during cryopreservation (25, 40). In addition, in vivo and clinical trials studies have shown the beneficial effects of oral administration of ascorbic acid in oligo-astheno-teratozoospermia. 

In these studies oral ascorbic acid supplementation can improve sperm concentration, motility and morphology (19, 25). However, in animal studies, the beneficial effects of ascorbic acid administration to different types of sperm media have remained controversial issue, which some studies have shown beneﬁcial effects on sperm functions and the others reporting not any protective impact (25).

## Conclusion

In summary, addition of ascorbic acid to the sperm processing medium from teratozoospermic samples may improve progressive motility, viability, and DNA integrity, but no acrosome reaction ability. However, further studies are required in order to use ascorbic acid and other antioxidants in the sperm processing medium in teratozoospermia to optimize the sperm quality. 
